# Integrated single-cell and bulk RNA-Seq analysis enhances prognostic accuracy of PD-1/PD-L1 immunotherapy response in lung adenocarcinoma through necroptotic anoikis gene signatures

**DOI:** 10.1038/s41598-024-61629-8

**Published:** 2024-05-13

**Authors:** Ping Sui, Xueping Liu, Cheng Zhong, Zhanming Sha

**Affiliations:** 1 Department of Oncology, The Affiliated Yantai Yuhuangding Hospital of Qingdao University, Yantai, 264000 Shandong China; 2 Institute of Transfusion Medicine and Immunology, Mannheim Institute for Innate Immunoscience (MI3), Medical Faculty Mannheim, University of Heidelberg, Heidelberg, Germany; 3 Department of Pulmonary and Critical Care Medicine, The Affiliated Yantai Yuhuangding Hospital of Qingdao University, Yantai, 264000 Shandong China; 4 Jiangmen Hospital of Traditional Chinese Medicine Affiliated to Jinan University, Jiangmen, 52900 China; 5 Department of Anesthesiology, Shandong Provincial Third Hospital, Jinan, 250031 China

**Keywords:** Cancer, Cell biology, Genetics, Immunology, Diseases

## Abstract

In addition to presenting significant diagnostic and treatment challenges, lung adenocarcinoma (LUAD) is the most common form of lung cancer. Using scRNA-Seq and bulk RNA-Seq data, we identify three genes referred to as HMR, FAM83A, and KRT6A these genes are related to necroptotic anoikis-related gene expression. Initial validation, conducted on the GSE50081 dataset, demonstrated the model's ability to categorize LUAD patients into high-risk and low-risk groups with significant survival differences. This model was further applied to predict responses to PD-1/PD-L1 blockade therapies, utilizing the IMvigor210 and GSE78220 cohorts, and showed strong correlation with patient outcomes, highlighting its potential in personalized immunotherapy. Further, LUAD cell lines were analyzed using quantitative PCR (qPCR) and Western blot analysis to confirm their expression levels, further corroborating the model's relevance in LUAD pathophysiology. The mutation landscape of these genes was also explored, revealing their broad implication in various cancer types through a pan-cancer analysis. The study also delved into molecular subclustering, revealing distinct expression profiles and associations with different survival outcomes, emphasizing the model’s utility in precision oncology. Moreover, the diversity of immune cell infiltration, analyzed in relation to the necroptotic anoikis signature, suggested significant implications for immune evasion mechanisms in LUAD. While the findings present a promising stride towards personalized LUAD treatment, especially in immunotherapy, limitations such as the retrospective nature of the datasets and the need for larger sample sizes are acknowledged. Prospective clinical trials and further experimental research are essential to validate these findings and enhance the clinical applicability of our prognostic model.

## Introduction

Current- and ex-smokers, as well as non-smokers, are at high risk of lung adenocarcinoma (LUAD), the predominant histologic subtype of lung cancer. Accounting for about 40% of lung cancer cases^1^, LUAD has a bad prognosis with an only approximately 16% 5-year survival rates^2^. Patients with early-stage LUAD may be able to undergo standard surgery, but those with advanced LUAD will still face considerable challenges from conventional radiology and chemotherapy^3,4^. This critical situation has led to the development of immune checkpoint inhibitors, particularly those targeting PD-1 and PD-L1, have transformed the therapeutic landscape, offering hope for better outcomes^5^. However, the variability in response to these therapies underscores the necessity of identifying novel approaches and refining patient selection to enhance the efficacy of treatment in LUAD.

In recent research on the tumor microenvironment, cancer-associated fibroblasts (CAFs) have been identified as playing a pivotal role in various cancers^6^. These cells originate from normal fibroblasts, which are transformed under the influence of hormones and cytokines within the tumor microenvironment. GPX8 has been implicated in fostering an immunosuppressive tumor microenvironment, which contributes to the adverse prognosis observed in LUAD^7^.

Diverse forms of programmed cell death have long been focal points of tumor research. There are numerous factors making tumor progression possible, but necroptosis, mediated by RIP1 kinase and RIP3, is one of the most important. Necroptosis has a significant impact on tumor outcome^8^. Additionally, necroptosis regulatory factors and their transcriptional changes could markedly impact cancer prognosis in various solid tumors^9–11^. Found by Seifert el al, relationship exists between necroptosis and tumor microenvironment (TME) signaling through the RIP1/RIP3 pathway^12^. Additionally, several studies have demonstrated that necroptosis can also promote tumor growth by recruiting inflammatory immune cells^13^.

Anoikis occurs when cells detach from the extracellular matrix (ECM), disrupting integrin ligation, a necessary function of tumor cells after detachment from the ECM^14^. Through both intrinsic and extrinsic pathways, various molecular markers including TNF-α, Bcl-2 and novel signal pathways induced by anoikis have been identified as a crucial factor in tumor progression^15–17^. Anoikis is uniquely capable of influencing cancer progression and metastatic spread due to its immune-related checkpoints, offering new immunotherapeutic approaches.

All mentioned evidence suggests a significant interplay between necroptotic anoikis in the context of lung adenocarcinoma (LUAD). To address these above challenges, our study initially focuses on unraveling the intricate interactions at the single-cell level to unveil the underlying mechanisms driving LUAD progression. Expanding our exploration to the transcriptomic landscape. Building on these results, we develop predictive models to assess immune infiltration and identify potential therapeutic targets. Ultimately, our aim is to enhance the treatment against lung cancer, particularly by improving the efficacy of PD-1 and PD-L1 checkpoint inhibitors, thus paving the way for more personalized and effective therapeutic strategies.

## Materials and methods

### Single cell and bulk RNA-seq data sources and processing

Lung carcinoma tissue samples, sourced from the Cancer Genome Atlas (TCGA) database (https://cancergenome.nih.gov/), were analyzed for gene expression patterns. This analysis incorporated vital clinical factors like patient's survival status, total lifespan post-diagnosis, demographic details (age and gender), and the grading of the lung carcinoma. Samples missing comprehensive survival data were omitted from the study. The TCGA database provided a wealth of information, including RNA sequencing transcriptome profiles (quantified as FPKM values) and in-depth clinicopathological characteristics from a cohort of 500 lung carcinoma cases and 59 healthy lung tissue samples. A thorough examination of genetic alterations in these lung carcinoma patients was undertaken, with a focus on identifying patterns in somatic mutations and copy number variations (CNVs). To further substantiate these findings, additional comparative analysis was carried out, involving 127 lung carcinoma specimens, retrieved from the GEO database with the accession of GSE50081. This comparative approach aimed to validate the initial observations and explore any potential genomic markers or trends unique to lung carcinoma.

Adenocarcinoma tissues from the GSE149655 and GSE162498 datasets were sequenced on the 10X Genomics platform using single-cell RNA sequencing (scRNA-seq). A comprehensive bioinformatics protocol was applied to the scRNA-seq data. Initially, we used the Seurat analytical package for preprocessing. As part of this project, a correlation assessment was conducted to determine relationships between sequencing depth, mitochondrial gene representation, and intracellular gene counts using the PercentageFeatureSet function for quantifying mitochondrial gene expression compared to total gene expression.

For a rigorous analytical framework, gene expression was filtered, retaining only those genes expressed in a minimum of 5 cells. Cell selection criteria were stringently applied, encompassing a range of gene expression (over 300 and under 5000 genes), a mitochondrial gene percentage below 10%, and a threshold of 1000 unique molecular identifiers (UMIs) per cell. Post-filtering, scRNA-seq data normalization was conducted via the LogNormalize method, laying the groundwork for accurate subsequent analyses.

### Determination and annotation of necroptotic anoikis-associated DEGs

Genes associated with anoikis were identified through comprehensive database searches in GeneCards [https://www.genecards.org/] and Harmonizome [https://maayanlab.cloud/Harmonizome/], using ‘Anoikis’ as the principal search parameter. This approach resulted in the identification of 640 genes related to anoikis and 67 genes connected to necroptosis. These genes were then rigorously screened, applying a relevance threshold of 0.4. An unsupervised clustering analysis, focusing on the expression patterns of these necroptotic anoikis-related genes, was performed using the consensusClusterPlus package in the R programming framework.

Necroptosis-related genes were extracted from a curated selection of existing scientific publications. A Pearson correlation examination was initiated to delineate the associations between genes governing overall anoikis and necroptosis, adhering to strict parameters: a Correlation Coefficient (Cor) exceeding 0.6 and a significance level (P-value) below 0.05. This meticulous process unveiled 25 genes distinctly linked to necroptotic anoikis. Further, a comparative analysis of these genes was undertaken. In this phase, the overall profile of differentially expressed genes (DEGs) was ascertained, guided by criteria of an absolute fold change surpassing 0.585 and a p-value under 0.05.

### Unsupervised clustering of necroptotic anoikis-associated differentially expressed genes

Cluster analysis devoid of supervision was executed utilizing the ConsensusClusterPlus package in R, leveraging the k-means algorithm, a mainstay in machine learning. This approach involves categorizing cases into distinct groups based on specific biomarkers or signatures indicative of certain biological states or processes. The expression patterns of these hallmark gene sets mirror these specific biological dynamics. After multiple sampling, the optimal k value was identified when the number of clusters k = 2, 3, 4, … 9. Analyzing variations in the Cumulative Distribution Function (CDF) curve areas by employing tools like the Item-Consensus plot and the Proportion of Ambiguous Clustering score, the determination of the ideal cluster quantity was achieved when the index was up to the approximate maximum. This led to the delineation of two separate clusters in the context of necroptotic anoikis, designated as cluster A and B. Following this, a comparative analysis of the Overall Survival (OS) rates between these clusters was conducted, employing Kaplan–Meier survival plots.

### Correlations between necroptotic anoikis-associated gene signature and clinical parameters

From the univariate Cox regression scrutiny of the intersected genes related to necroptotic and immune responses in lung cancer, P-values exceeding 0.01 were deemed statistically significant. Following this, a Cox regression construct, underpinned by the LASSO (Least Absolute Shrinkage and Selection Operator) approach, was developed. This model integrated genes with identified prognostic value from training cohorts within The Cancer Genome Atlas (TCGA). The prognostic coefficients for these genes were determined by implementing a tenfold cross-validation on the lambda values extracted from the ‘glmnet’ package.

This process led to the identification of a risk signature correlating with both necroptotic and immune response, effectively prognosticating patient outcomes in lung carcinoma. The risk score was computed using the formula:

$$Risk score ={\sum }_{i=1}^{n}Coefi * xi$$

On the basis of the median risk score, lung carcinoma patients were grouped into high-risk and low-risk categories. Kaplan–Meier analysis and log-rank tests were used to compare overall survival times between the groups. To evaluate the predictive accuracy of the gene signature, survival, survivalminer, and timeROC packages were used. A similar statistical method and formula were used in order to confirm the prognostic validity of this gene signature in lung carcinoma in the GEO cohort.

### Identifying a prognostic signature of necrotic anoikis subcluster and its response to chemotherapy drugs

Using single-sample gene set enrichment analysis (ssGSEA), we examined the differences in immune cell infiltration between populations at high and low risk. To determine the efficacy of riskscore as a predictor of immunotherapy response, a comparative analysis of immune checkpoint expression levels was performed. Additionally, the oncoPredict R package was used to identify disparities in the effectiveness of targeted therapies among different patient cohorts.

### Function enrichment analysis

ClusterProfiler R package's Gene Set Enrichment Analysis (GSEA) feature was used to analyze function enrichment. As part of this analysis, MSigDB was used as the source of the gene set ‘c2.cp.kegg.v7.4.symbols.gmt’ from the C2 gene set. It was found that the top five hallmark gene sets in each subgroup, prioritizing those with a p-value below 0.05, were related to functional pathway differences between these subtypes. Moreover, functional pathway differences between these subtypes were determined by gene set variation analysis. To determine whether differentially expressed genes (DEGs) have gene ontology (GO) and Kyoto Encyclopedia of Genes and Genomes (KEGG) features, we used the ‘clusterProfiler’ R package, focusing on q-values less than 0.05 to determine statistical significance. To visually delineate the enriched KEGG pathways among the subtypes, heatmaps were meticulously crafted using the ggkegg function within the ggplot2 R package. This method involved mapping statistically significant KEGG pathways to their corresponding gene expression profiles^18–20^. The resulting heatmaps were then color-coded based on the degree of enrichment, providing a detailed and intuitive visualization that underscored the distinct biochemical pathways active in each subtype, essential for interpreting complex biological differences.

### Predictive nomogram development and validation

Evaluating the prognostic efficacy of a risk score for lung cancer involved both univariate and multivariate Cox regression analyses, coordinated with clinical factors like age and sex. These analyses identified independent factors for prognosis, adhering to a p-value significance criterion under 0.05. An interactive graphical representation, a nomogram, was developed using the ‘regplot’ package in R, designed to forecast overall survival probabilities at 1, 3, and 5 years for individuals with lung cancer.

The accuracy of these prognostic estimations was validated through the construction of calibration curves. To appraise the predictive significance and discriminatory power for 3, 5, and 10-year survival intervals, Kaplan–Meier survival plots were employed along with time-dependent receiver operating characteristic (ROC) analyses. Further, a decision curve analysis (DCA) was executed to affirm the predictive utility and clinical applicability of the nomogram in the context of lung cancer prognosis.

### Immune infiltration analysis

The ESTIMATE algorithm, which utilizes gene expression signatures to approximate the proportion of immune and stromal cells within tumor samples, was employed to calculate the ImmuneScore, StromalScore, and ESTIMATEScore for predicting tumor purity. These scores were correlated with DRG expression using Spearman's method, with results depicted in scatter plots that include p-values and correlation coefficients.

For the assessment of tumor microenvironment (TME) cell infiltration, we employed the single-sample gene set enrichment analysis (ssGSEA) approach using the “GSVA” R package. This method facilitates the evaluation of immune cell presence based on gene expression profiles indicative of specific immune cells. Using Spearman correlation analysis, we investigated the associations between key genes and various immune cell types, streamlining the focus to include key immune populations such as activated B cells, CD4+ and CD8+ T cells, dendritic cells, and natural killer cells among others, providing a comprehensive yet concise assessment of immune landscape within the tumor microenvironment.

### Process of epigenetic mutation data

Somatic alteration data for the TCGA-LUAD cohort were acquired from the TCGA database, facilitating an in-depth analysis of genetic variations. Tumor Mutational Burden (TMB) was meticulously defined as the total count of somatic, coding, base substitution, and insertion-deletion mutations per megabase of the genome sequenced. This calculation included non-synonymous and frameshift indels, adhering to a stringent 5% detection threshold to ensure precision. For the quantitative assessment of somatic non-synonymous point mutations within individual samples, the “maftools” R package was employed. This package not only provides comprehensive tools for the analysis, visualization, and summarization of mutation annotation format (MAF) files but also supports comparative studies and co-occurrence analysis. All analyses were performed using R version 4.1.3 (14/12/2023), providing a reliable and consistent computational environment.

### Cell culture

The lung cancer cell lines A549, along with normal lung fibroblast cells (MRC-5), were sourced from the American Type Culture Collection (ATCC). To prevent bacterial growth, a culture medium composed of Dulbecco’s Modified Eagle Medium (DMEM, Gibco, USA) enriched with 10% fetal bovine serum (FBS) and supplemented with 100 g/mL of a 1% penicillin–streptomycin solution was utilized. Cell cultures were maintained in an incubator set at 37 °C with a 5% humidity level, ensuring optimal conditions for cellular proliferation and health, which are critical for the validity of lung cancer research experiments.

### RNA isolation followed by quantitative reverse transcription-polymerase chain reaction (qRT-PCR) analysis

Quantitative real-time PCR (qRT-PCR) was utilized to evaluate gene expression levels in the human lung adenocarcinoma cell lines A549, as well as in MRC-5 normal lung fibroblasts. Total RNA was extracted using Trizol reagent (ThermoFisher, 15596026), and its purity and concentration were assessed using a NanoDrop 2000 spectrophotometer. Reverse transcription and qRT-PCR were conducted in accordance with the protocols provided with the TSK301 Reverse Transcription System Kit (Masterbio TSK301M).

The SYBR Green RT-qPCR Master Mix, essential for qRT-PCR, was supplied by Tsingke Biotechnology Co., Ltd. (Beijing, China). Primers targeting specific genes were designed and synthesized by GeneWIZ Bioengineering Co. in Suzhou. The PCR conditions included a denaturation step at 95 °C for 10 s, annealing at 60 °C for 30 s, and an elongation phase at 60 °C for 30 s. Glyceraldehyde 3-phosphate dehydrogenase (GAPDH) served as an internal control, and gene expression levels were normalized against it for quantitative assessments.

Cell cultures of A549 and MRC-5 lines were grown in DMEM (Gibco, USA) enriched with 10% fetal bovine serum (ABW, AB-FBS-1050S, Uruguay) and 100 g/mL penicillin–streptomycin (Invitrogen, Carlsbad, CA, USA). Cultivation occurred in a humidified atmosphere containing 5% CO2 at 37 °C, adhering to standard protocols. Cells were used for experimental purposes when they reached approximately 80% confluence during the logarithmic phase of growth. The study details specific primer sequences for each investigated gene, which include:

For KRT6A: Forward primer: ACCAGACCTTGCCGTTCATTAT, Reverse primer: TGACGTGGGAGTTGTGGATG.

For HMMR: Forward primer: GCTTGAGGTGTAGATGTGTCC, Reverse primer: CCCACGGGGCAAGATTTGAA.

For FAM83A: Forward primer: GCAAAACAGGGAAGAGTGTTCAT, Reverse primer: TAAGCCAACTCCAAGCCTGA.

### Western blotting

In this lung cancer investigation, Western blot analysis was conducted with proteins extracted from human lung adenocarcinoma cell lines (A549) and normal lung fibroblast cells (MRC-5). Protein extraction entailed lysing cells in RIPA buffer supplemented (Beyotime, Shanghai, China) with a PMSF protease inhibitor (CoWin Biosciences, Jiangsu, China) at a 1:100 ratio. Total protein was quantified using a bicinchoninic acid (BCA) protein assay kit (Thermo Scientific, Guangzhou, China). After quantification, each protein sample, measuring 10 μg, was loaded onto a 10% sodium dodecyl sulfate polyacrylamide gel electrophoresis (SDS-PAGE). Following electrophoresis, proteins were transferred to a 0.45 μm polyvinylidene difluoride membrane. These membranes were initially incubated with primary antibodies overnight at 4 °C, targeting KRT6A (BS, 1:1000), HMMR (BS, 1:1000), FAM83A (BS, 1:1000), and GAPDH (CST, 1:1000) as a standard reference. Protein band visualization was achieved using a chemiluminescence imaging system (Beijing, China) and an ECL chromogenic substrate kit (Thermo Fisher Scientific, Guangzhou, China).

### Statistical analysis

In this research, version 4.3.1 of the R software environment was utilized for rigorous data processing, analysis, and visualization. The Kaplan–Meier method, facilitated by the ‘survival’ package in R, was employed to assess survival data, along with log-rank test computations for survival analysis. LASSO regression, integrating cross-validation techniques, was performed using the ‘glmnet’ package. Receiver operating characteristic curves (ROCs) were generated in conjunction with the survival package, utilizing the ‘survminer’ package. For data following a Gaussian distribution, the Student’s *t*-test was applied, while the Wilcoxon rank-sum test was used for datasets deviating from normal distribution. Comparative statistical assessments between two distinct datasets involved methods such as the *t*-test and the Mann–Whitney *U* test. For multi-group comparisons, one-way analysis of variance (ANOVA) was the chosen method. Data visualization was achieved with ‘ggpubr’ and ‘ggplot2’, which are well-regarded R packages for producing high-quality graphical representations. A p-value of 0.05 was established as the criterion for statistical significance across all comparative evaluations, barring specific exceptions.

## Results

### ScRNA-Seq analysis of necroptotic anoikis genes in LUAD

**Figure 1:**
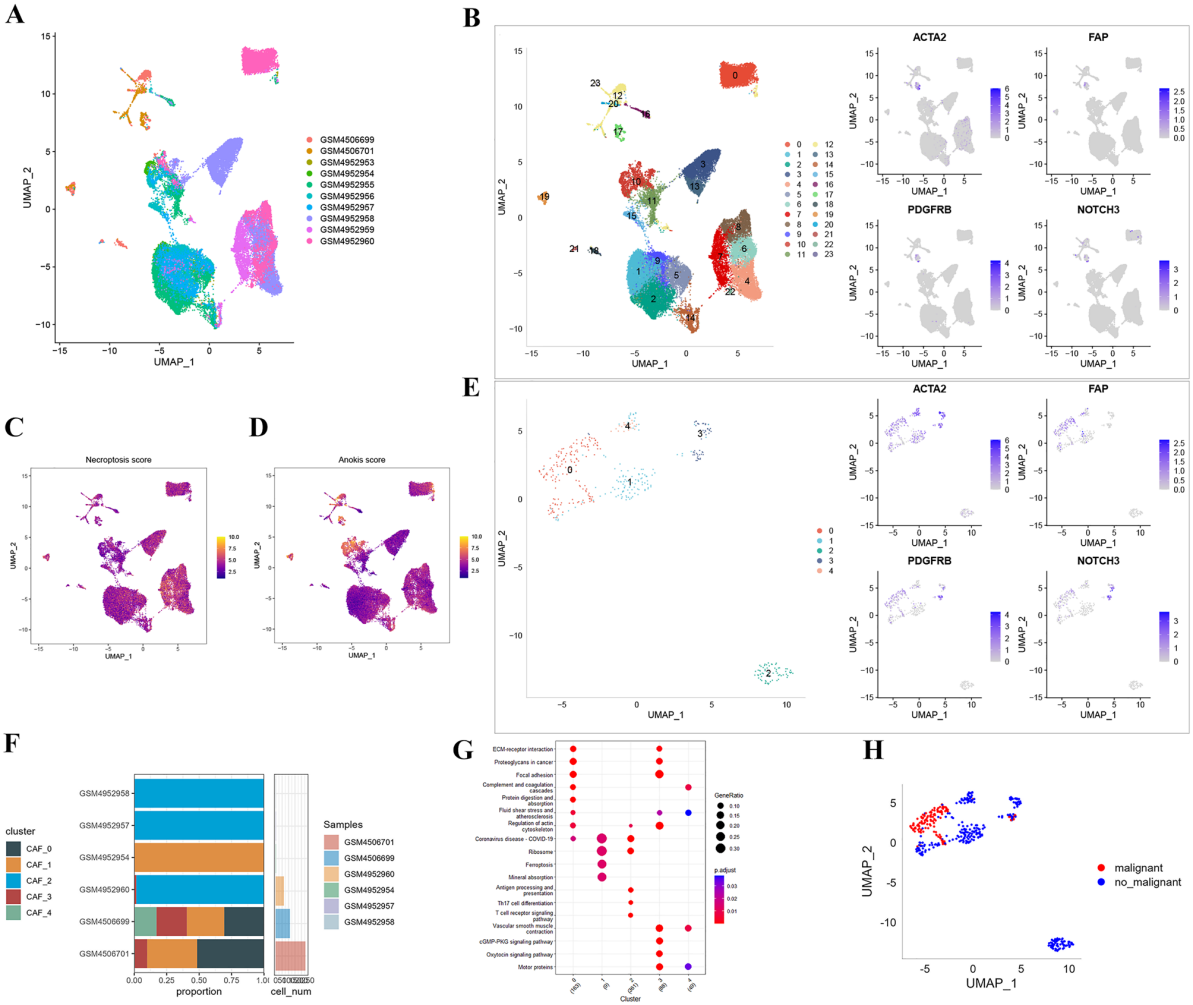
Comprehensive single-cell RNA sequencing analysis of lung adenocarcinoma tissue samples. (**A**) Uniform Manifold Approximation and Projection (UMAP) visualization depicting the heterogeneity of lung adenocarcinoma samples, each color representing a unique sample from the GEO dataset. (**B**) UMAP plot showing discrete cell populations based on expression profiles of cancer-associated fibroblast (CAF) marker genes ACTA2, FAP, PDGFRB, and NOTCH3. To the right, expression density plots for each marker gene across the UMAP coordinates. (**C**) Necroptosis score distribution across lung adenocarcinoma samples overlayed on UMAP coordinates. (**D**) Anoikis score distribution across lung adenocarcinoma samples overlayed on UMAP coordinates. (**E**) Dimensionality reduction clustering analysis depicting the presence of distinct clusters based on the expression of ACTA2, FAP, PDGFRB, and NOTCH3 marker genes. (**F**) Bar plot representing the proportion of different CAF clusters within individual lung adenocarcinoma samples from the GEO dataset. (**G**) Bubble plot illustrating the enriched pathways and biological processes across identified clusters, with bubble size representing the strength of association. KEGG pathway information was utilized for pathway enrichment analysis. (**H**) UMAP plot differentiating malignant from non-malignant cells based on gene expression signatures.

The gene expression profiles related to necroptotic anoikis in lung adenocarcinoma (LUAD) were meticulously evaluated. Clustering analysis, based on data from 10 patients in the GEO database (GSE149655 and GSE162498), was performed (as shown in Fig. 1A,B). This analysis involved assessing the association between two programmed cell death pathways and identified clusters by computing a “necroptosis score” and “anoikis score” (Fig. 1C,D). The role of cancer-associated fibroblasts (CAFs) in the immune microenvironment was acknowledged by selecting marker genes ACTA2, FAP, PDGFRB, and NOTCH3^21–23^, aiding in dimensionality reduction for the clustering analysis, which identified five CAF clusters (Fig. 1E).

The study also analyzed the proportion of each CAF subtype in various samples (Fig. 1F). Pathway analysis revealed functional differences among clusters, with ECM-receptor interaction and focal adhesion being prominent in certain clusters, potentially contributing to anoikis resistance (Fig. 1G). UMAP clustering differentiated malignant from non-malignant cells (Fig. 1H).

The relationship between the counts of unique molecular identifiers (UMIs) and mRNAs was investigated, showing a strong association, but no clear correlation with mitochondrial gene content (Supplementary Fig. 1A). Pre- and post-quality assurance data were visualized using violin plots (Supplementary Fig. 1B,C). Principal component analysis (PCA) indicated the exclusion of the 10th principal component from further analysis, given its statistical significance (Supplementary Fig. 1D,E).

To further explore necroptotic anoikis-related signatures, the study detailed the differential gene expression across the identified clusters (Supplementary Fig. 2A). Variations in gene expression among these clusters were quantified, providing a deeper understanding of the diverse gene expression patterns related to necroptotic anoikis in LUAD. In Supplementary Fig. 2B, we showed the top five marker genes among these identified clusters, which illustrated the difference in signature gene expression. Furthermore, we next explored various genes across different cell clusters in Supplementary Fig. 2C. These results clearly indicated there were significant differences among the clusters we categorized.

### Mutation landscape of necroptotic anoikis genes in LUAD

**Figure 2:**
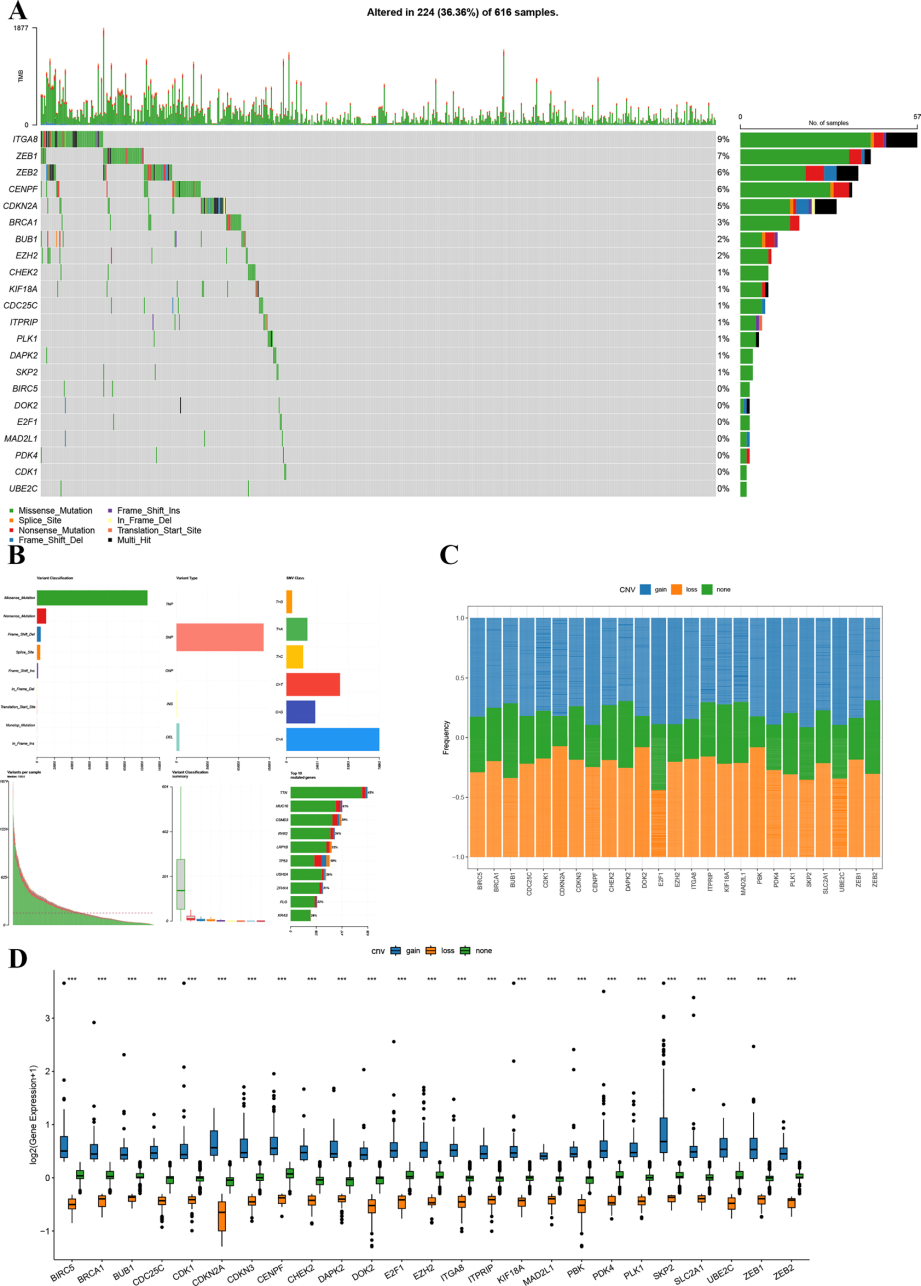
Overview of necrotic anoikis-related gene alterations in lung adenocarcinoma. (**A**) OncoPrint visualization detailing the frequency and types of genetic alterations for necrotic anoikis-related genes in a lung adenocarcinoma cohort, with mutations such as missense, frameshift insertion, and deletion marked across samples (top panel). The right panel quantifies the proportion of lung adenocarcinoma samples exhibiting specific genetic alterations. (**B**) The distribution of genetic alterations for each necrotic anoikis-related gene is represented in a stacked bar chart, with distinct colors indicating the type of alteration (middle panel). (**C**) A frequency plot delineates the incidence of copy number variations (CNVs) across the cohort, identifying gains and losses in these genes (lower left panel). (**D**) Differential gene expression of necrotic anoikis-related genes is illustrated via boxplots, with each data point representing expression variation in individual lung adenocarcinoma samples (bottom panel). The visualizations were crafted using the R packages ggplot2 for general plotting and ComplexHeatmap, which provides capabilities for more advanced visual representations. The analysis and visualization were performed in RStudio (version 2023), which can be downloaded from https://posit.co/download/rstudio-desktop/.

The mutation analysis of necroptotic anoikis genes in lung adenocarcinoma (LUAD) revealed significant genetic alterations within a subset of the cohort, as derived from the GEO dataset. An OncoPrint visual representation highlighted these mutations in 224 out of 616 samples (36.36%), underscoring the prevalence of genetic aberrations (Fig. 2A). Notable genes with frequent alterations included ITGA8 (9%), ZEB1 (7%), ZEB2 (6%), among others, exhibiting a range of mutation types from missense to frameshift deletions. These genes were proved to relate with programed cell deaths and cancer immune in previous studies^24–26^.

The study further examined the distribution of these mutations across various LUAD patient samples, uncovering a heterogeneous pattern of genetic changes. Boxplots illustrating the expression levels of these genes revealed significant variability across patients, suggesting potential links to diverse clinical outcomes (Fig. 2B). A frequency plot was used to detail mutation frequencies for each gene (Fig. 2C), coupled with boxplots that outlined the expression levels of these genes (Fig. 2D). This approach shed light on the complex mutation landscape of necroptotic anoikis regulatory genes in LUAD, laying the groundwork for subsequent investigations into their functional roles and therapeutic implications.

### Construction of necroptotic anoikis genes-related molecular subclusters

Using the ConsensusClusterPlus package, Consistent Clustering Analysis (CCA) was conducted using a set of necroptotic anoikis-related genes identified in lung adenocarcinoma (LUAD). On the basis of gene expression levels, LUAD patients were grouped into distinct groups. Differential gene expression analysis further delineated two molecular subclusters within the cohort, centered around key necroptotic anoikis genes (Supplementary Fig. 3A). These subclusters exhibited unique expression profiles, as evidenced in the heatmap, with significant gene expression differences outlined (Supplementary Fig. 3B).

Principal Component Analysis (PCA) was employed to discern specific necroptotic anoikis-related patterns, leading to the stratification of patients into two clusters (Supplementary Fig. 3C,D). The determination of the optimal number of subclusters was guided by a delta area plot (Supplementary Fig. 3E). Survival analysis indicated that patients in cluster A had a more favorable outcome compared to those in cluster B, underscoring the potential prognostic significance of these gene signatures (Supplementary Fig. 3F).

### Identification of DEGs in clusters and function enrichment analysis

In the analysis presented in Supplementary Fig. 4A, GSVA enrichment highlighted distinct biological functions in two identified subgroups. Cluster A predominantly featured pathways related to vascular smooth muscle activity and calcium signal transduction, whereas Cluster B was characterized by enrichment in cellular proliferation processes, including cell cycle regulation, DNA replication, and RNA degradation. This analysis also identified 510 genes that exhibited differential expression between Clusters A and B. Supplementary Fig. 4B further illustrates that these differentially expressed genes are integral to critical tumor-related processes such as division of nuclear material, segregation of chromosomes, and specifically, the segregation within nuclear chromosomes.

Additionally, as shown in Supplementary Fig. 4C, KEGG pathway analysis underscored a significant enrichment of pathways related to oncogenesis and immune response in Cluster B, highlighting mechanisms involved in cell cycle progression, DNA replication, and the pathogenic impact of Human T-cell leukemia virus 1.

### Development and immune analysis of the necroptotic anoikis signature

**Figure 3:**
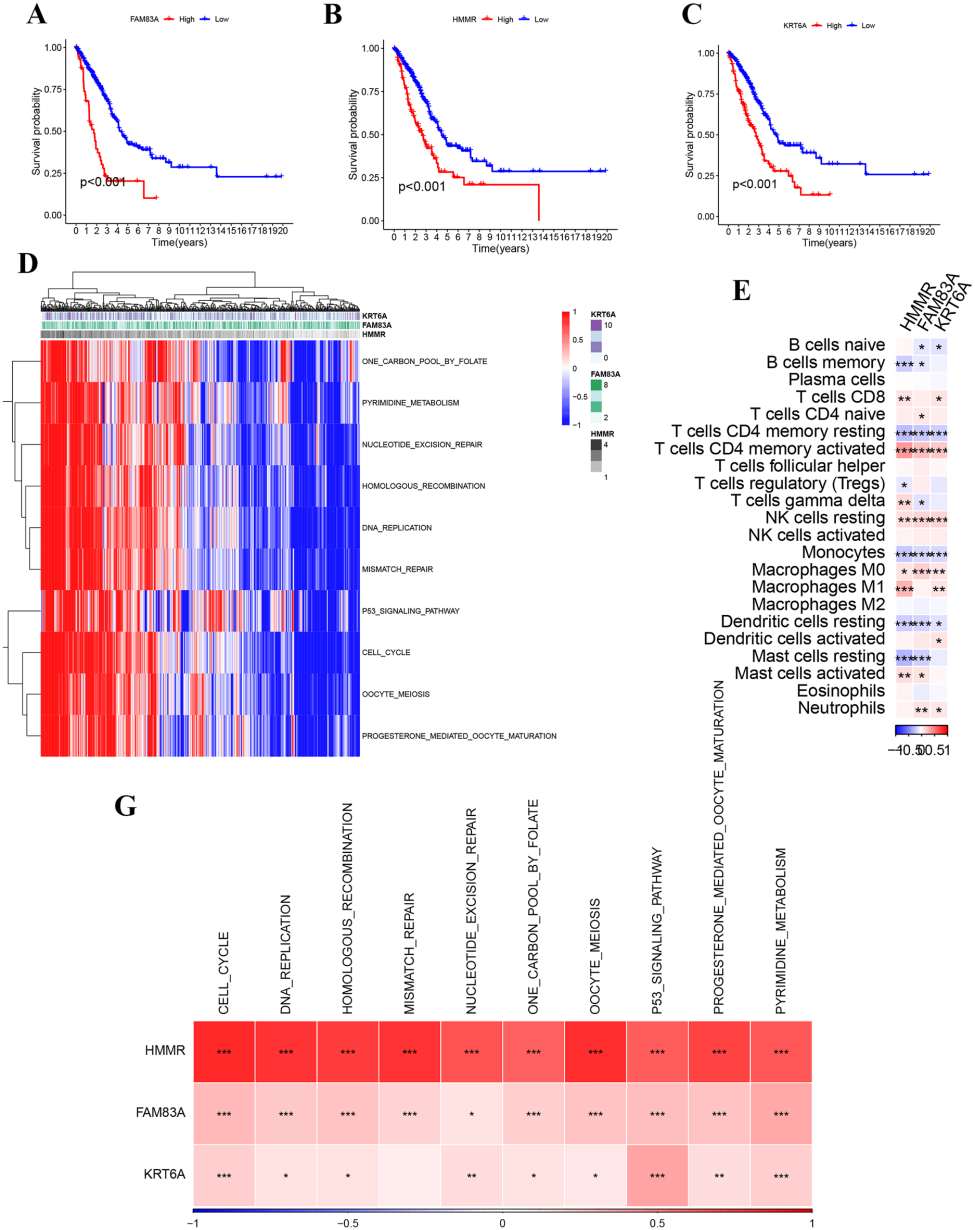
Novel signature model genes and their prognostic and immunological correlations in lung adenocarcinoma. (**A**–**C**) Kaplan–Meier survival curves for lung adenocarcinoma patients stratified by expression levels of the novel signature model genes FAM83A, HMMR, and KRT6A, showing a significant correlation between high gene expression and reduced survival probability (p < 0.001). (**D**) Heatmap detailing the expression patterns of the novel signature model genes alongside their interaction with critical biological pathways such as cell cycle regulation and p53 signaling, highlighting potential mechanisms influencing tumor behavior. The visualizations were crafted using the R packages ggplot2 for general plotting and ComplexHeatmap, which provides capabilities for more advanced visual representations. The analysis and visualization were performed in RStudio (version 2023), which can be downloaded from https://posit.co/download/rstudio-desktop/. (**E**) Correlation heatmap displaying the interactions between the novel signature genes and various immune cell infiltrates within the tumor microenvironment, including T cells, B cells, and myeloid cell populations, suggesting their potential influence on immune evasion and response. The visualizations were crafted using the R packages ggplot2 for general plotting and CIBERSORT, which provides capabilities for more advanced visual representations. The analysis and visualization were performed in RStudio (version 2023), which can be downloaded from https://posit.co/download/rstudio-desktop/. . (**F**) Scatter plot analysis indicating the correlation between HMMR expression and the gene signatures of immune cells, with the trend line providing a visual representation of the predictive relationship. (**G**) Summary bar graph illustrating the degree of correlation between the signature genes and key oncogenic pathways, with the color gradient representing the strength of the correlation from negative (blue) to positive (red).

Analysis of patient survival related to the three genes demonstrated a notable association: elevated expression of these genes corresponded with lower overall survival rates, as depicted in Fig. 3A–C. Figure 3D presents heatmaps illustrating variances in gene expression profiles and associated biological pathways, particularly those involving DNA replication and repair processes. Further, Fig. 3E delineates the disparity in immune cell infiltration among different types of immune cells between groups with high and low gene expression. This suggests that divergent immune evasion strategies might contribute to the observed variations in infiltration levels of resting NK cells and M0 macrophages. Moreover, a scatter plot analysis (Fig. 3F) demonstrated a correlation between gene expression and biomarker prevalence, highlighting the complex interplay between these genes and tumor biology. Figure 3G further detailed the correlation strengths between the genes and key biological processes, accentuating the multifaceted nature of their influence on cellular functions. These mentioned results revealed the association between immune environment and both the necroptotic anoikis signature.

Our next step was to use multivariate Cox regression analysis and LASSO regression analysis to classify LUAD patients into low- and high-risk groups based on their overall survival (OS). This approach involved selecting three genes for the construction of the prognostic signature. The stratification results for the two groups are presented in Supplementary Fig. 5A,B. Our model showed excellent stratification capability in training cohort, internal cohort and external cohort (respectively Supplementary Fig. 5C–K). The K-M curves in Supplementary Fig. 5L,M further demonstrated the model divided patients into two distinct groups statistically significantly (p < 0.05). Additionally, as illustrated in Supplementary Fig. 5N, our model effectively differentiated patient subgroups according to survival probability, underscoring its potential utility in predicting clinical outcomes and guiding precision medicine strategies.

### Predictive nomogram development and validation

**Figure 4:**
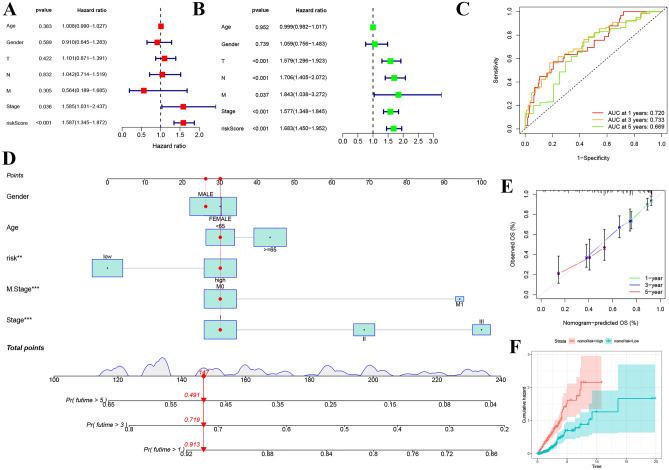
Multifaceted prognostic evaluation using novel signature model in lung adenocarcinoma. (**A**) Forest plot displaying the univariate Cox proportional hazards analysis, indicating hazard ratios for age, gender, tumor stage, and risk score, with the risk score showing a significant association with patient outcomes (p < 0.001). (**B**) Forest plot from multivariate Cox analysis, confirming the independent prognostic value of tumor stage and risk score in lung adenocarcinoma after adjusting for other clinical factors. (**C**) Receiver operating characteristic (ROC) curves illustrating the discriminative performance of the novel signature model at 1, 3, and 5 years, with areas under the curve (AUCs) demonstrating the model’s predictive accuracy. (**D**) A nomogram integrating clinical variables and the novel risk score, offering a quantitative tool for predicting the probability of survival at specified time points. (**E**) Calibration plots comparing the nomogram-predicted overall survival (OS) with actual observed OS at 1, 3, and 5 years, assessing the predictive accuracy of the nomogram. (**F**) Box plots of nomogram components, scoring individual clinical parameters and risk score, facilitating personalized risk assessment. (**G**) The Kaplan–Meier curve stratifies patients into high- and low-risk groups based on the median risk score derived from the novel signature model, with significant differences in survival outcomes.

In the context of lung adenocarcinoma (LUAD), clinical characteristics are pivotal in prognostic determination. To evaluate the prognostic significance of a developed risk score, multivariable Cox regression analyses were utilized. Forest plots (Fig. 4A,B) illustrated the hazard ratios associated with various clinical factors, such as age, gender, tumor stage, and the calculated risk score. The risk score emerged as a significant prognostic indicator (p < 0.001), with higher scores correlating with increased hazard ratios. With these results, we constructed a predictive nomogram as shown in Fig. 4D.

ROC curve analyses validated the constructed nomogram, yielding areas under the curve (AUCs) of 0.720 after 1 year, 0.733 after 3 years, and 0.669 after 5 years (Fig. 4C). Clearly, our nomogram performed well in terms of prediction.

Overall survival for 1, 3, and 5 years was concordant with nomogram-predicted OS (Fig. 4E). Using Kaplan–Meier survival curves, patients are classified as high-risk or low-risk according to their median risk score. The prognostic relevance of the nomogram was confirmed by the fact that LUAD patients in the high-risk group had significantly lower overall survival than those in the low-risk group (Fig. 4F). These findings indicate that the nomogram can be applied to precision treatment strategies for patients with LUAD, emphasizing its predictive capability.

### GSVA, GSEA and ssGSEA of novel signature

**Figure 5:**
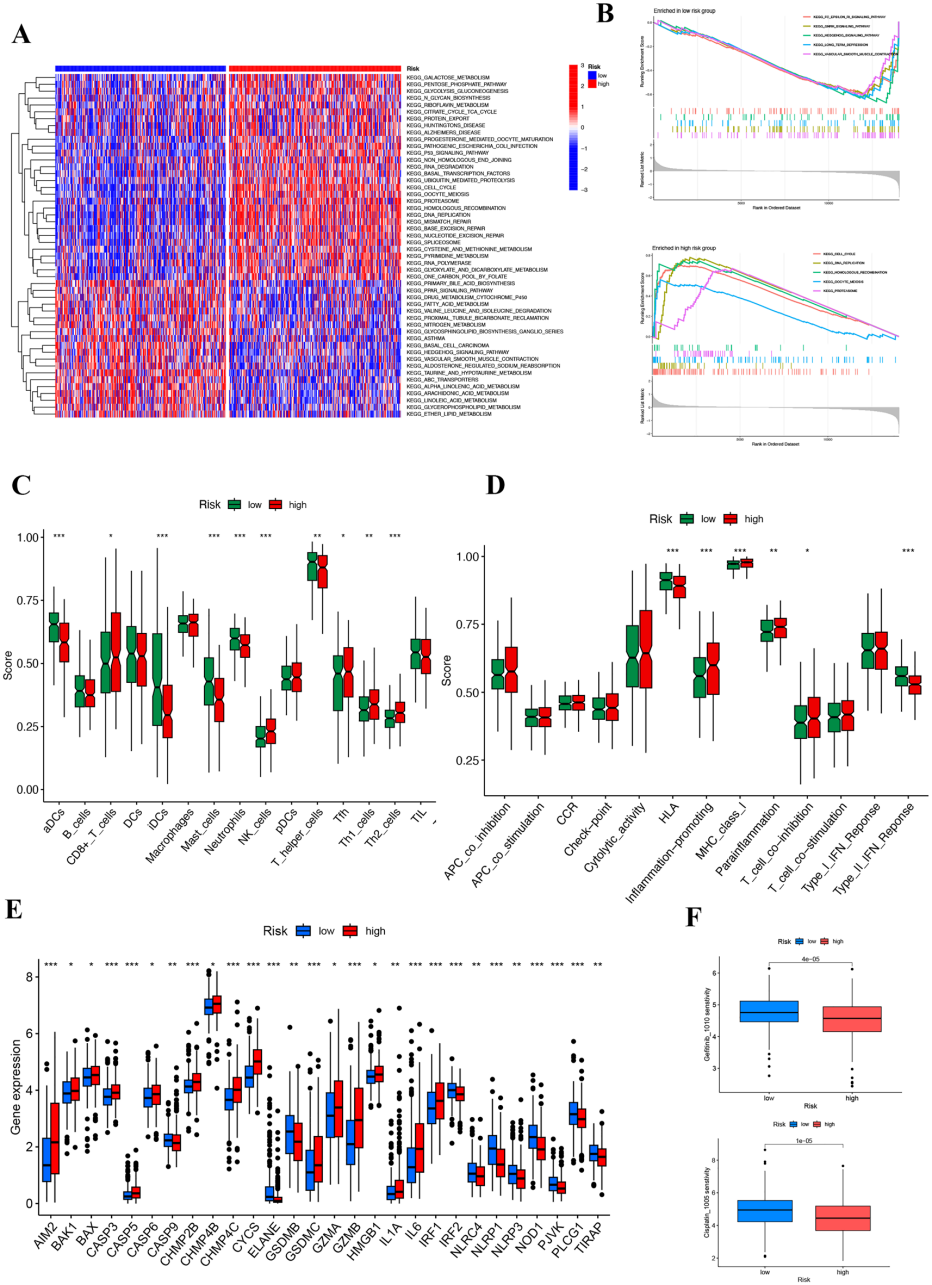
Integrated analysis of the necrotic anoikis-related risk signature in lung adenocarcinoma (LUAD). (**A**) Heatmap displaying gene expression profiles across LUAD samples, clustered by low and high necrotic anoikis-related risk scores. The expression levels are color-coded, with red representing upregulation and blue representing downregulation. The side bar indicates the risk category of each sample. The visualizations were crafted using the R packages ggplot2 for general plotting and ComplexHeatmap, which provides capabilities for more advanced visual representations. The analysis and visualization were performed in RStudio (version 2023), which can be downloaded from https://posit.co/download/rstudio-desktop/. (**B**) Gene set enrichment analysis (GSEA) for low and high-risk groups. The top plot shows the enriched pathways in the low-risk group, while the bottom plot displays those in the high-risk group, with the normalized enrichment score (NES) plotted along the x-axis. (**C**) Box plots comparing the infiltration scores of various immune cells between low and high-risk groups, demonstrating significant differences in the immune landscape associated with the risk categories. (**D**) Comparison of selected immune-related functional activities between low and high-risk groups, highlighting differences in checkpoint regulation, cytotoxic activity, and other immune functions. (**E**) Box plots detailing the expression of immune checkpoint genes, comparing low and high-risk groups, indicating potential implications for immunotherapy responsiveness. (**F**) Box plots represent the sensitivity to chemotherapy agents, comparing the gene expression variability and its association with treatment response in low versus high-risk LUAD groups, indicating the potential for personalized therapy based on the risk signature.

In the gene set variation analysis (GSVA) and gene set enrichment analysis (GSEA) of a novel gene signature in lung adenocarcinoma (LUAD), distinct expression patterns related to immune cell fractions were observed (Fig. 5A). The heatmap in Fig. 5A delineates differential expression profiles across samples, with red indicating upregulation and blue denoting downregulation within the context of identified pathways.

Enrichment plots for both low and high-risk groups are presented in Fig. 5B, demonstrating distinct pathway activations in these cohorts. Notably, in our low-risk group, identified pathways including the GNRH signaling pathway, whereas the high-risk group showed enrichment in pathways such as cell cycle and DNA replication. Additionally, the analysis of enrichment scores across immune-related gene sets revealed significant disparities between low and high-risk groups (Fig. 5C,D), with the latter showing enhanced activation of key oncogenic pathways.

The proportion of immune cells is positively correlated with a lower risk score, according to statistical analysis. Based on single-sample GSEA (ssGSEA), we determined the amount and type of immune cells infiltrating the tumor microenvironment, and we found that low-risk and high-risk groups expressed different levels of checkpoint molecules. Upregulated expression was observed in the low-risk group, hinting at potential benefits from immune therapy (Fig. 5E). Lastly, for both Gefitinib and Cisplatin, which are important chemotherapy drugs^27,28^, it demonstrated a higher sensitivity for low-risk patients, corroborating its potential as a prognostic indicator (Fig. 5F).

### Prediction of immunotherapy response to drugs and PD/PDL1 treatment

**Figure 6:**
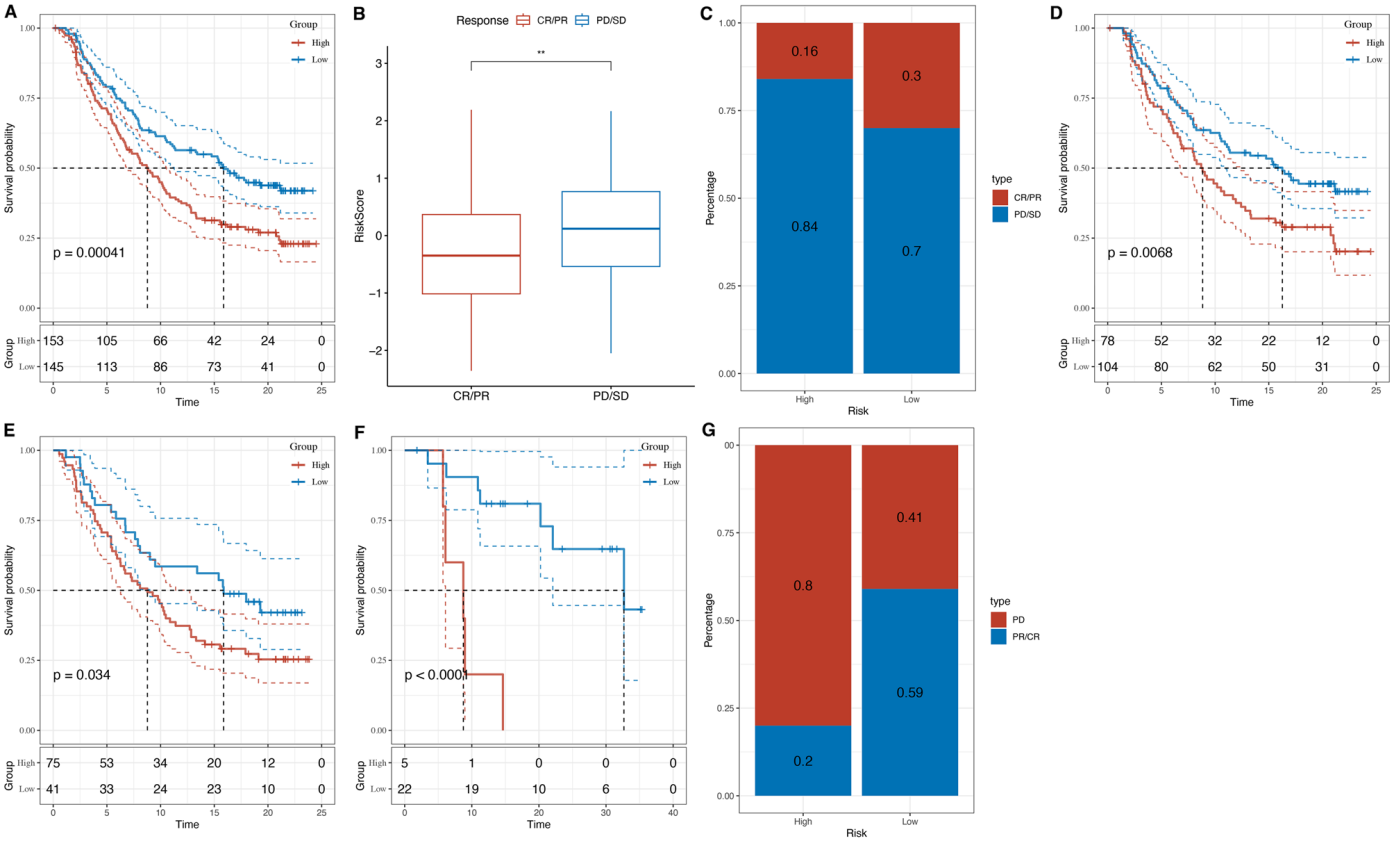
Prognostic value of necrotic anoikis-related risk score in patient outcomes and response to therapy in lung adenocarcinoma (LUAD). (**A**) Kaplan–Meier survival curves depicting the difference in survival probability between high and low-risk groups based on the necrotic anoikis-related risk score, with significant separation indicating prognostic relevance (p = 0.00041). (**B**) Box plot illustrating risk scores in relation to patient response to therapy, categorizing complete response/partial response (CR/PR) and progressive disease/stable disease (PD/SD), indicating a higher risk score is associated with poorer response. (**C**) Stacked bar graph showing the proportion of patients with CR/PR versus PD/SD in high and low-risk groups, demonstrating a higher percentage of PD/SD in the high-risk category. (**D**) Kaplan–Meier analysis for a separate patient cohort, confirming the prognostic significance of the risk score (p = 0.0068). (**E**) Another Kaplan–Meier survival curve for an additional patient subset, further validating the risk score’s prognostic impact (p = 0.034). (**F**) Kaplan–Meier curve depicting long-term survival probability, reinforcing the risk score’s predictive capacity for patient outcomes (p < 0.0001). (**G**) Proportions of PD versus CR/PR in high and low-risk groups from another patient cohort, highlighting a greater tendency for PD in the high-risk group.

A predictive model, incorporating four critical genes, was constructed and subsequently verified across three separate lung adenocarcinoma (LUAD) patient cohorts, encompassing both the IMvigor210 and GSE78220 datasets. This prognostic framework initially segregated LUAD patients into two risk strata: high and low, as demonstrated in Fig. 6A within the IMvigor210 cohort. A marked distinction in survival likelihood (P < 0.0001) was observed between these risk groups.

Within the IMvigor210 cohort, diverse therapeutic responses to PD-1/PD-L1 checkpoint inhibition were recorded, ranging from complete responses (CR) and partial responses (PR) to stable disease (SD) and progressive disease (PD). Patients exhibiting PD/SD presented higher risk scores compared to those with CR/PR, as shown in Fig. 6B. The incidence of SD/PD was notably higher in the high-risk group, and this group was also linked with significantly poorer outcomes, as indicated in Fig. 6C.

Consistent validation of the model was seen across additional cohorts, including the IMvigor210 dataset. Figure 6D specifically represents the responses of stage I + II patients within the IMvigor210 cohort to PD-1/PD-L1 inhibition therapy. Figure 6D demonstrates consistent validation of the predictive model in this subgroup, with all p-values being less than 0.05. Similarly, Fig. 6E represents the responses of stage III + IV patients within the IMvigor210 cohort, reinforcing the model's credibility for this stage.

Moreover, Fig. 6F furnishes insights into the therapeutic responses of patients within the GSE78220 cohort, specifically delineating the responses of stage III + IV patients to PD-1/PD-L1 inhibition therapy within the GSE78220 dataset. Figure 6F underscores the dependability of the model and its extension of applicability to a distinct dataset. Furthermore, Fig. 6G elucidates the proportions of PD versus CR/PR in high and low-risk groups from another patient cohort, accentuating a pronounced predilection for PD in the high-risk group.

### Validation of novel genes expression levels in mRNA and protein levels in cell lines

**Figure 7:**
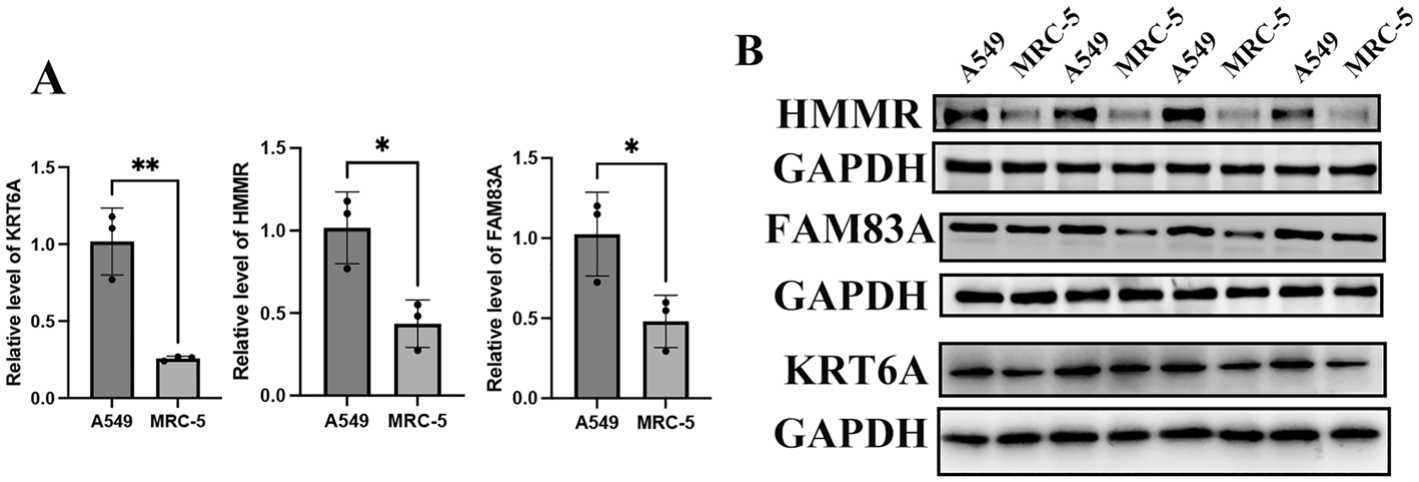
External validation. (**A**) The mRNA level (relative to GAPDH) of KRT6A, HMMR, and FAM83A in lung adenocarcinoma cells compared to normal pulmonary cells. (P < 0.05). (**B**) The protein expression of KRT6A, HMMR, and FAM83A in lung adenocarcinoma cells compared to normal pulmonary cells.

To ascertain the expression levels of these three candidate genes in lung adenocarcinoma, we conducted a meticulous quantitative PCR (qPCR) analysis on a range of cell lines. This qRT-PCR analysis (Fig. 7A,B) revealed a distinct expression profile in lung adenocarcinoma cells, showcasing variations in gene expression when compared to normal pulmonary cells. These differences in expression patterns may be indicative of the genes' roles in the oncogenic processes of lung adenocarcinoma.

Complementing the qPCR analysis, Western blot analysis in Fig. 7C,D was employed to evaluate the protein expression levels of the genes KRT6A, HMMR, and FAM83A. This protein analysis provided a deeper insight into the cellular mechanisms at play, revealing that the protein expression of these genes in lung adenocarcinoma cells was notably different from that in normal lung cells. Such variations in protein expression levels could reflect the functional implications of these genes in the pathophysiology and progression of lung adenocarcinoma. This dual approach of qRT-PCR and Western blot analysis offers a comprehensive understanding of both the transcriptional and translational modifications associated with these genes in the context of lung cancer.

### Pan cancer of model genes and risk signature

**Figure 8:**
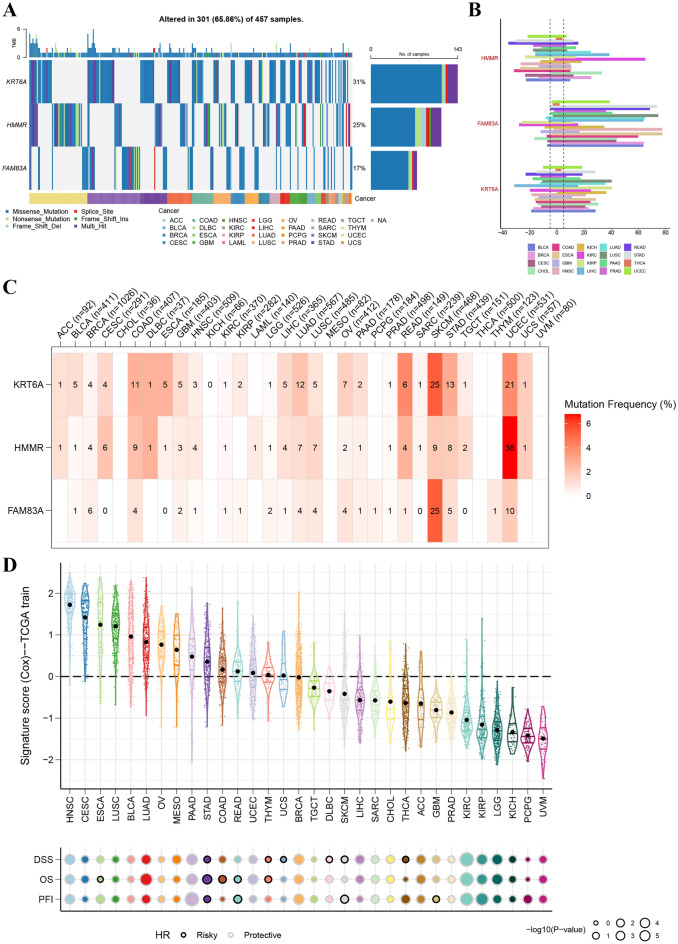
Pan-cancer analysis of necrotic anoikis-related model genes KRT6A, HMMR, and FAM83A. (**A**) OncoPrint visualization demonstrating the alterations of KRT6A, HMMR, and FAM83A across 457 pan-cancer samples, with a summary stack bar indicating the percentage of samples affected by mutations in each gene. (**B**) Horizontal bar graph depicting the distribution and variance of mutation types for each gene across different cancer types, providing a mutation landscape across the pan-cancer spectrum. (**C**) Heatmap showing the mutation frequency of each model gene in different cancer types, with the intensity of red correlating to higher mutation frequencies, offering a clear visual of gene-specific mutation prevalence. The visualizations were crafted using the R packages ggplot2 for general plotting and ComplexHeatmap, which provides capabilities for more advanced visual representations. The analysis and visualization were performed in RStudio (version 2023), which can be downloaded from https://posit.co/download/rstudio-desktop/. (**D**) Violin plots representing the signature scores (derived from Cox regression analysis) for each cancer type, with the colors indicating different clinical outcomes such as disease-specific survival (DSS), overall survival (OS), and progression-free interval (PFI). Below, a bubble chart illustrates the hazard ratios (HR), with color coding denoting risk (risky or protective) and size corresponding to the significance level (− log10 p-value).

To broaden the scope of our research and validate the widespread applicability of our model, an expanded investigation was conducted to evaluate its validation and predictive performance in a pan-cancer cohort. Figure 8A,B showcase the mutations of KRT6A, HMMR, and FAM83A across 28 different cancer types, detailing the specific mutation types. These findings suggest that the impact of the identified genes extends beyond lung adenocarcinoma (LUAD), influencing a wide array of cancer types. The mutation frequency heatmap in Fig. 8C further corroborates the mutations of these three genes across the pan-cancer spectrum.

The analysis indicates that the distribution of the necroptotic anoikis-related RiskScore varies across cancer types, further highlighting the unique characteristics of our lung cancer-derived gene signature. Additionally, the association of this signature with key outcomes such as disease-specific survival, overall survival, and progression-free intervals further underscores its potential relevance and applicability across various oncological contexts. Notably, while this signature was initially derived from lung cancer-specific genes, its effectiveness across different cancers underlines its broad relevance, reinforcing the value of our initial findings. This supports the initial hypothesis of the model's credibility and applicability, as evidenced in Fig. 8D.

## Discussion

In this study, we elucidated the relationship between lung adenocarcinoma (LUAD) and two forms of programmed cell death: necroptotic anoikis. Given the rapidly progressive development of LUAD, it has been difficult to improve the patients’ prognosis through singular targeted therapies. Through revealing necroptotic anoikis-related gene expressions, we identified three key genes significantly correlated with LUAD outcomes, by which, we developed a predictive model, providing important tools for risk assessment and validation for patients. The model not only aids in understanding disease progression but also provides guidance for personalized therapeutic strategies.

Among malignant tumors, lung adenocarcinoma has the highest mortality and morbidity rates in China and even in world^29^. Given the limited efficacy associated with standard treatments, there is a pressing need for the advancement of immunotherapeutic approaches in the treatment of LUAD. As the most common type of lung cancer^30^, there is a critical need to investigate molecular markers related to diagnosis and prognosis in LUAD patients. Related to necroptosis, which played an important role in tumorigenesis and metastasis^31^, the evasion of anoikis been identified as a significant factor facilitating tumor invasion and progression^32^. Therefore, the signature of necroptotic anoikis-related genes were identified to affect outcomes and guide for precision treatment in LUAD, especially for immunotherapy.

The aim of this study was to evaluate the relevance of necroptotic anoikis-related genes in LUAD by analyzing both single-cell and bulk RNA sequencing data. To further analyze the pool of individual cells, we identified and isolated cancer-associated fibroblasts (CAFs) after RNA-seq quality control and normalization procedures.

Cancer-associated fibroblasts (CAFs), a predominant cell type within the tumor microenvironment (TME), have gained considerable attention due to their multifaceted roles in tumor progression and therapeutic responses. These fibroblasts not only influence the TME through inflammation modulation, extracellular matrix (ECM) remodeling, and immune cell interactions but also through processes such as angiogenesis and immune evasion. CAFs can exhibit both pro-tumorigenic activities, including T-cell exclusion and enhanced cancer cell survival, and anti-tumorigenic effects, such as the production of a dense collagen matrix that may inhibit tumor growth and metastasis. Interestingly, certain CAF subtypes are associated with improved therapy outcomes, highlighting their potential as therapeutic targets. However, clinical trials broadly targeting the tumor stroma have yet to achieve significant success, suggesting the complexity of CAF functions. This complexity is further illustrated by single-cell RNA sequencing (scRNA-seq) studies that have unveiled significant heterogeneity among CAFs in various cancer contexts, including breast cancer, pancreatic ductal adenocarcinoma, and lung cancer. This heterogeneity and the dual functional roles of CAFs emphasize the need for refined therapeutic strategies that precisely target these fibroblasts within the TME.

To explore the mechanisms of the necroptotic anoikis-based molecular subtype, we employed a consensus clustering approach, which enabled the classification of the LUAD patients into two distinct subtypes, stratified according to their expression profiles of necroptotic anoikis-related genes. Patients classified in Cluster A exhibited a more favorable prognosis compared to those in Cluster B. By analyzing gene set variation (GSVA), the functional differences between the two clusters were uncovered, with cluster A showing a notable enrichment in immune pathways, including arachidonic acid metabolism which might be an apoptotic signal that regulates programmed cell death processes^33^. The observed differences between the two clusters further revealed a significant association between necroptotic anoikis and immune environments in LUAD.

The necroptotic anoikis-related-related gene signature was further defined through univariate Cox and LASSO Cox regressions using HMMR, FAM83A, and KRT6A. The AUC values of this signature were 0.720, 0.733, and 0.669 respectively across all cohorts. Based on these gene signatures, and clinical parameters, we developed a nomogram that provides a comprehensive forecast of patient outcomes. The calibration curve in our study confirms that the nomogram is clinically robust in its prognosis. Differentially expressed genes between the two subtypes were identified as 510 in total. These DEGs significantly enriched in pathways related to immune response and tumor growth based on GO and KEGG analyses.

The tumor microenvironment (TME) in lung adenocarcinoma constitutes a complex interplay of immune cells, stromal cells, and tumor cells^34^, each crucial in modulating tumor progression and influencing clinical outcomes. Solid tumors with higher ratios of CD163+ macrophages, non-classical monocytes, and intermediate monocytes have poorer survival rates, while solid tumors with an increased proportion of mast cells have a prolonged survival rate^35^. Additionally, a greater number of B cells is significantly associated with better overall survival^36^. Through our comprehensive analysis between the necroptotic anoikis-related signature and immune environment for LUAD, the patients in low-risk group demonstrated higher expression thus could receive a better outcome for standard chemotherapy and specially for immunotherapy like PD-1/PD-L1 blockade.

Subsequently, our model demonstrated robustness in stratifying LUAD patients into low- and high-risk groups for the overall survival (OS) across three independent cohorts. Notably, our analysis indicates that patients with poorer outcomes exhibit higher RiskScores, which also correlate with reduced sensitivity to PD-1/PD-L1 blockade. High-risk groups identified by our model consistently presented with worse prognoses. The results showed the potential utility of our model in guiding immunotherapy for lung adenocarcinoma.

Apart from analysis on single-cell sequencing results, we demonstrated that the role of these three genes utilizing RT-qPCR and Western blotting. The cell experiments further confirmed the value of our neoteric anoikis-related signature.

Finally, a comprehensive pan-cancer analysis in TCGA cohort was performed to conduct the expression and mutational landscape of HMMR, FAM83A, and KRT6A, aiming to understand their roles across diverse cancer types. Our study found that not only in LUAD patients, but the mutations of these genes were also observed in various tumors, which has been reported in previous studies^37–39^. These fundings mentioned still require to be proved in the future.

HMMR, also known as CD168 and located on chromosome 5, plays a multifaceted role in cancer progression. It is involved in cell cycle regulation, promotes macrophage polarization, and facilitates epithelial-to-mesenchymal transition^40^. Moreover, HMMR's interaction with low molecular weight hyaluronic acid (HA) fragments notably enhances immune cell recruitment and exacerbates patient prognosis by activating tumor microenvironment dynamics and influencing pathways such as CD44 expression^41^. FAM83A, found on chromosome 8q24 and overexpressed in various cancers, impacts lung adenocarcinoma through the Wnt/β-catenin signaling pathway and is linked to PD-L1 expression, affecting immune responses in cancer therapy^42^. Additionally, FAM83A’s expression is further promoted by the antisense RNA FAM83A-AS1, enhancing lung cancer cell growth^43^. KRT6A, a type II keratin involved in the epidermalization of squamous epithelium, plays a critical role in cell migration and cancer metastasis. Its elevated levels in lung adenocarcinoma are associated with poor prognosis, primarily through mechanisms that promote the epithelial–mesenchymal transition^44^.

While our findings contribute valuable insights, it’s important to acknowledge the limitations of this study, which include: Firstly, all of our data were gathered from the GEO database, which may impact the comprehensiveness of the proposed model as well as the clarity of potential mechanisms. Secondly, the relatively small sample size of lung adenocarcinoma cases in the GEO database may have affected the statistical significance of some findings. Lastly, further experimental research and clinical research are required to verify our conclusions.

In the context of personalized lung cancer treatment, the riskScore we've introduced becomes paramount. By categorizing patients based on this score, we can precisely stratify patients into different risk groups, each potentially requiring a distinct therapeutic approach. For instance, individuals with higher riskScores may require more aggressive treatment modalities or novel agents that specifically target genes identified in our study. On the other hand, patients with lower riskScores might derive greater benefit from immunotherapies, particularly due to the increased expression of immune checkpoint molecules. This kind of precision in patient stratification ensures that treatment modalities are not ‘one-size-fits-all’, but rather tailored to each patient's unique genomic profile. As the medical community advances toward more individualized care, such riskScore analyses will be pivotal in optimizing therapeutic strategies for lung cancer patients.

## Conclusion

We have developed a novel prognostic signature with remarkable predictive accuracy for lung adenocarcinoma (LUAD) prognosis. As a result of this study, necroptotic anoikis is highlighted as an important component of LUAD pathogenesis and provided insight into clinical decision-making and therapeutic strategies for lung adenocarcinoma management.

### Supplementary Information

Supplementary Information.

Supplementary Figure 1.

Supplementary Figure 2.

Supplementary Figure 3.

Supplementary Figure 4.

Supplementary Figure 5.

Supplementary Legends.

## Data Availability

Data generated or used in this study are available from the corresponding authors upon reasonable request.
