# Supplementary figures and images for: Integrated single-cell and bulk RNA-Seq analysis enhances prognostic accuracy of PD-1/PD-L1 immunotherapy response in lung adenocarcinoma through necroptotic anoikis gene signatures
Source: Sci Rep. 2024 May 13;14:10873. doi: 10.1038/s41598-024-61629-8 (PMC11091124; doi:10.1038/s41598-024-61629-8)

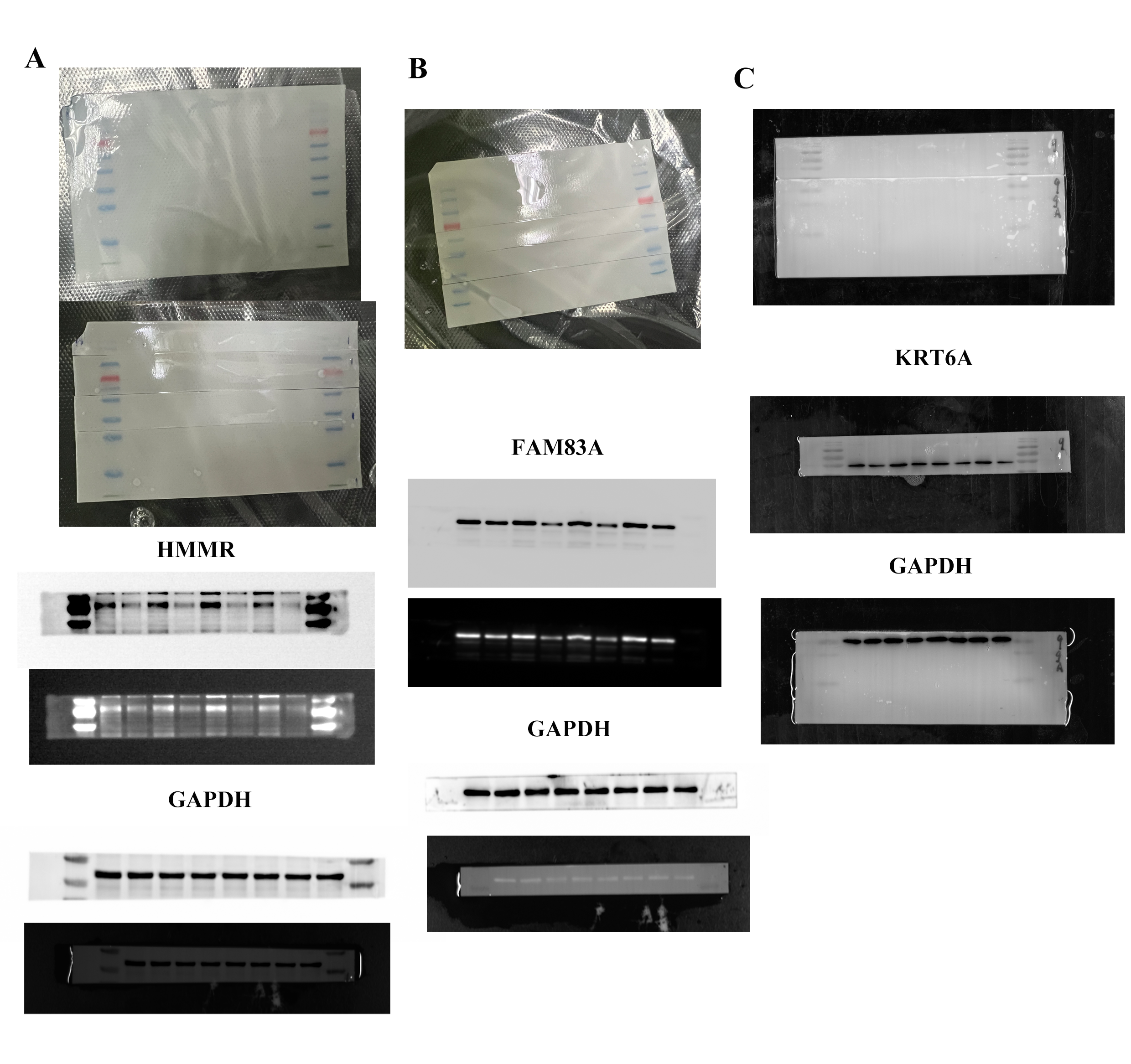

Supplement: Supplementary file 1 — Supplementary Information. [file 41598_2024_61629_MOESM1_ESM.tif]

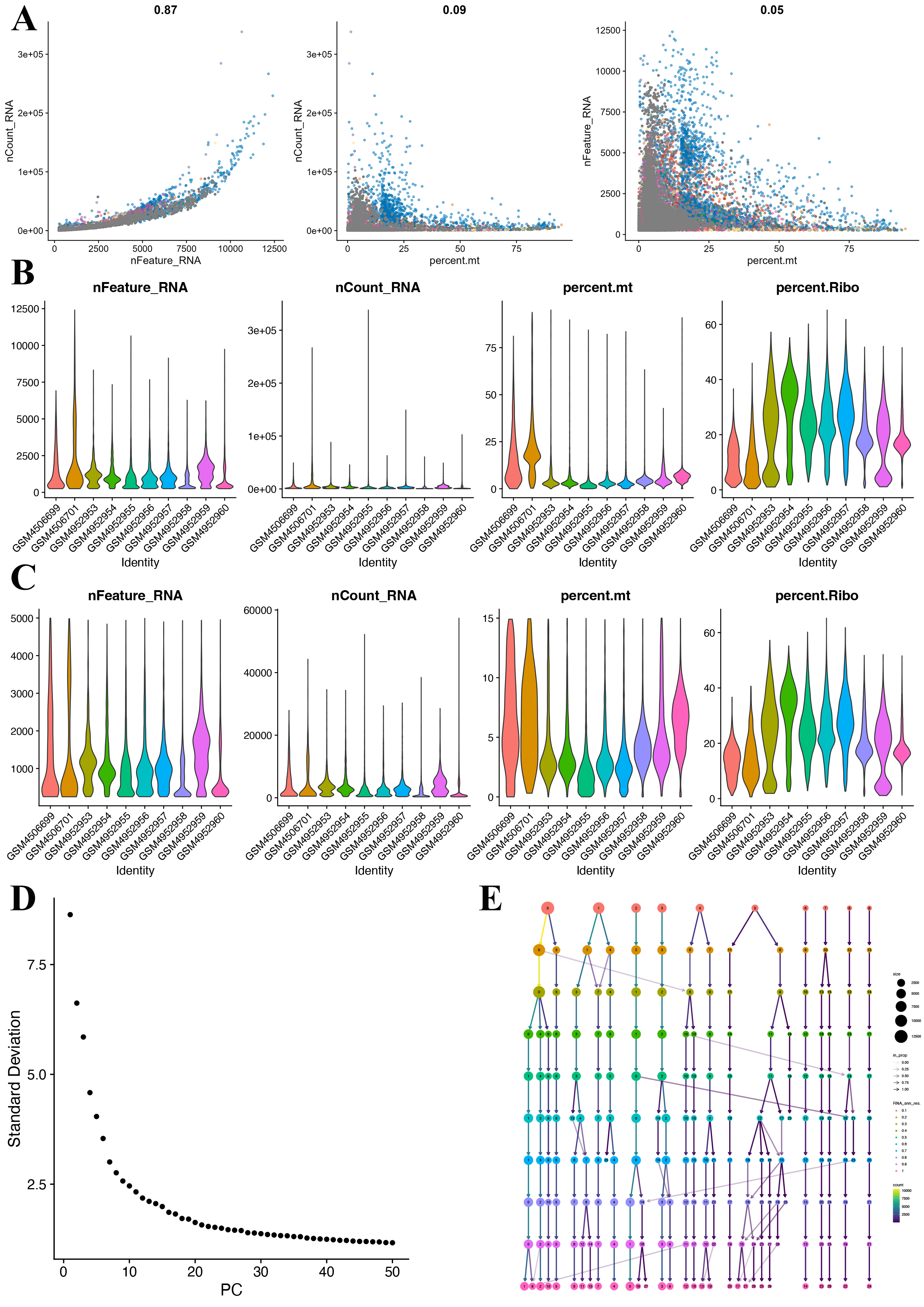

Supplement: Supplementary file 2 — Supplementary Figure 1. [file 41598_2024_61629_MOESM2_ESM.tif]

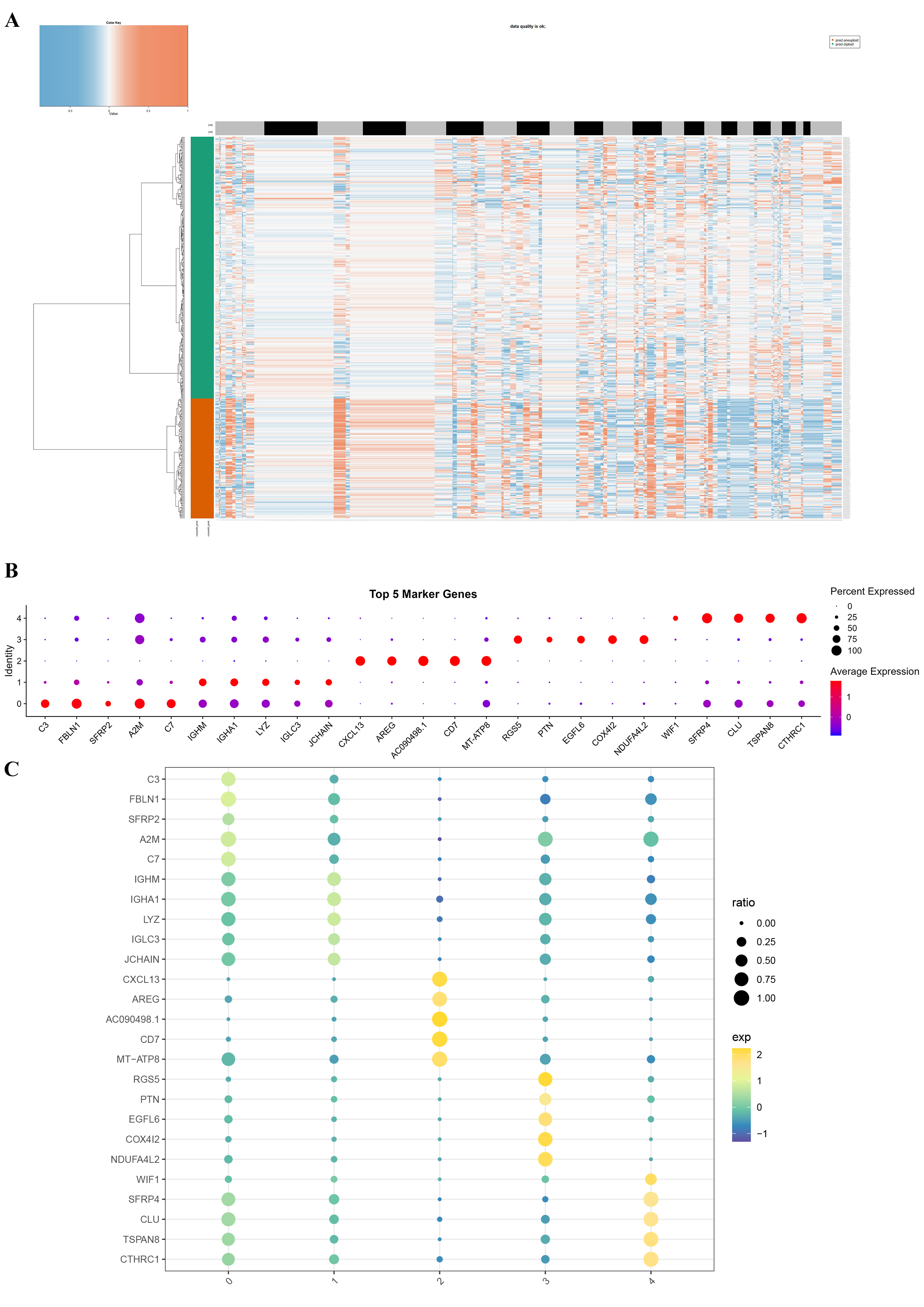

Supplement: Supplementary file 3 — Supplementary Figure 2. [file 41598_2024_61629_MOESM3_ESM.tif]

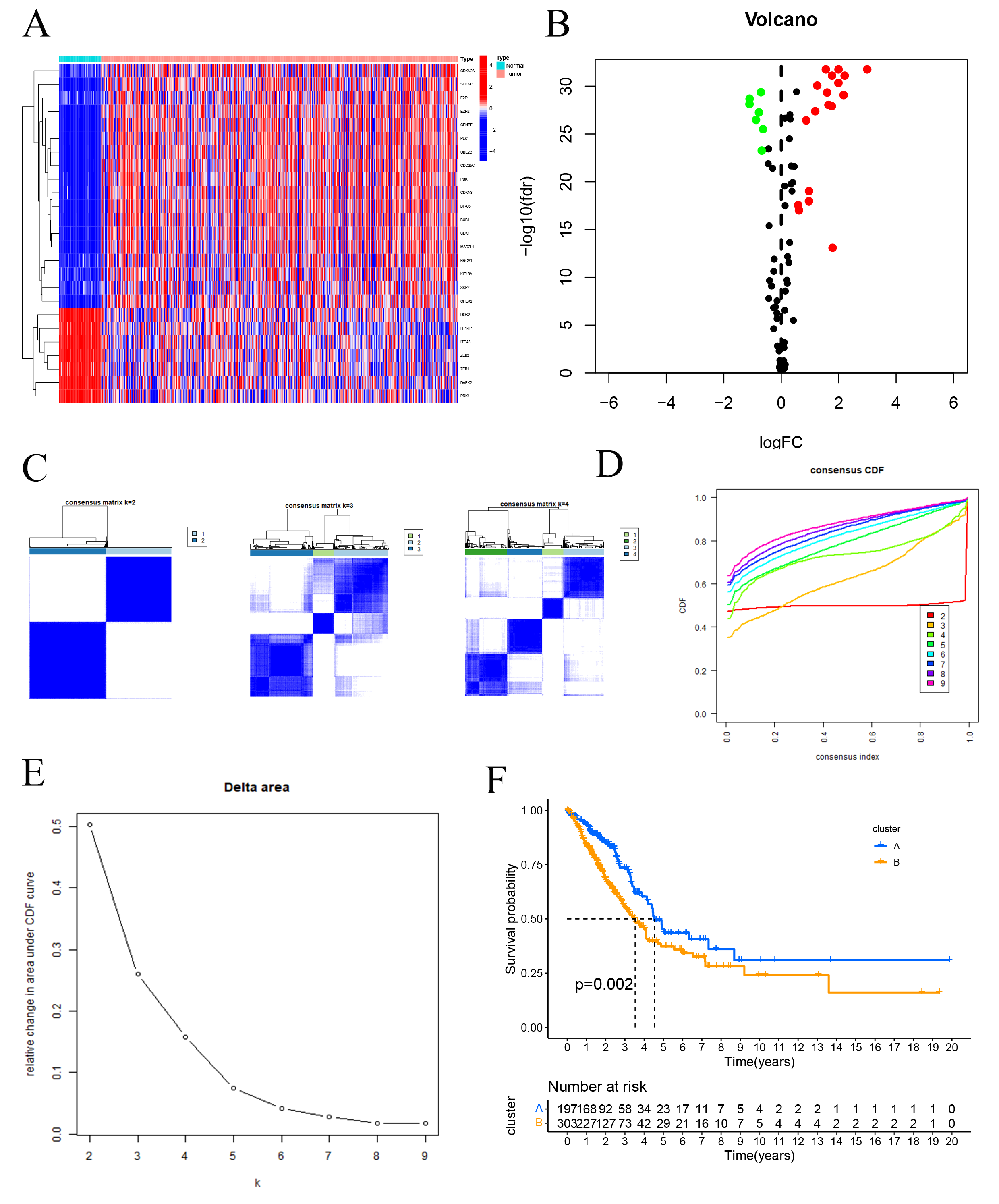

Supplement: Supplementary file 4 — Supplementary Figure 3. [file 41598_2024_61629_MOESM4_ESM.tif]

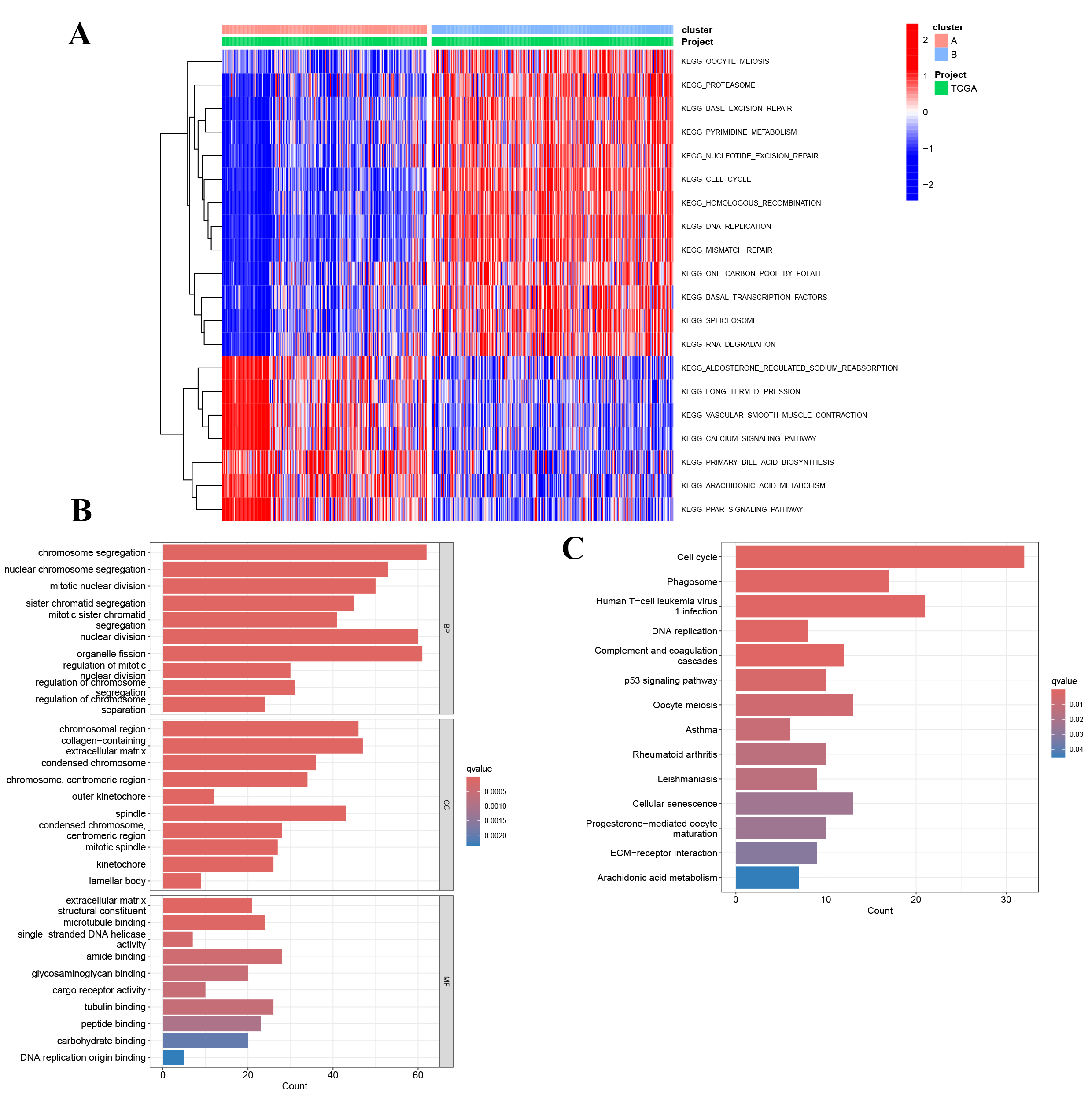

Supplement: Supplementary file 5 — Supplementary Figure 4. [file 41598_2024_61629_MOESM5_ESM.tif]

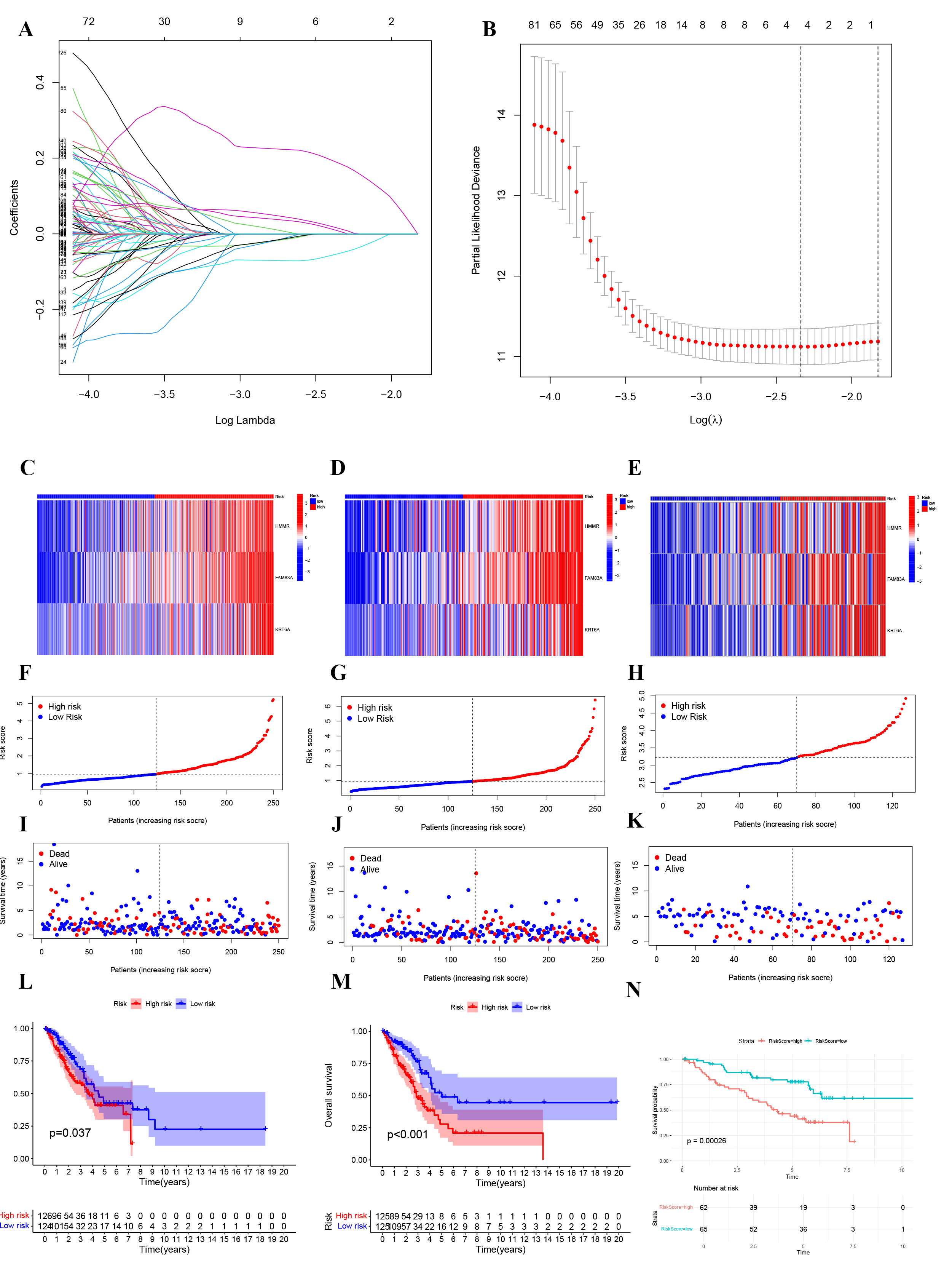

Supplement: Supplementary file 6 — Supplementary Figure 5. [file 41598_2024_61629_MOESM6_ESM.tif]
