# Supplementary material for: Integrated single-cell and bulk RNA-Seq analysis enhances prognostic accuracy of PD-1/PD-L1 immunotherapy response in lung adenocarcinoma through necroptotic anoikis gene signatures
Source: Sci Rep. 2024 May 13;14:10873. doi: 10.1038/s41598-024-61629-8 (PMC11091124; doi:10.1038/s41598-024-61629-8)
Supplement: Supplementary file 7 — Supplementary Legends. [file 41598_2024_61629_MOESM7_ESM.docx]

**Supplementary Figure 1: Quality Control and Feature Selection in Single-Cell RNA Sequencing Data of Lung Adenocarcinoma.** **(A)** Scatter plots illustrating the quality control metrics for single-cell RNA sequencing data, including the number of genes detected per cell (nFeature_RNA), the total counts per cell (nCount_RNA), the percentage of mitochondrial gene counts (percent.mt), and the percentage of ribosomal gene counts (percent.Ribo). Each point represents a single cell, with the correlation between these metrics shown. **(B)** Violin plots representing the distribution of quality control metrics across different cell identities in the dataset, showcasing the variability in gene detection, total counts, mitochondrial gene percentages, and ribosomal gene percentages. **(C)** Additional violin plots for a subset of cells, focusing on the same quality control metrics, providing a detailed view of the distribution of these features within specific cell populations. **(D)** Elbow plot depicting the standard deviation of the principal components (PCs), used to determine the number of significant PCs to retain for downstream analysis. **(E)** Dot plot showing the correlation of different principal components with the quality control metrics, with the size of the dots proportional to the strength of the correlation and the color indicating the specific metric.

**Supplementary Figure 2: Gene Expression Profiling and Marker Gene Analysis in Lung Adenocarcinoma Single-Cell RNA Sequencing Data.** **(A)** Heatmap with hierarchical clustering showing the expression levels of genes across single cells from lung adenocarcinoma samples. The color gradient from blue to red represents the normalized expression levels, with blue indicating lower and red indicating higher expression. Clusters of cells are differentiated by color coding on the dendrogram. **(B)** Dot plot representing the top 5 marker genes for each identified cell cluster. Dot size reflects the percentage of cells expressing the gene, and the color intensity represents the average expression level of that gene within the cluster. **(C)** Bubble chart illustrating the expression levels and ratio of various genes across different cell clusters. The size of the bubbles indicates the proportion of cells within a cluster expressing a given gene (ratio), while the color gradient shows the average expression level (exp).

**Supplementary Figure 3: Clustering Analysis and Differential Expression in Lung Adenocarcinoma.** **(A)** Heatmap displaying the differential gene expression across various clusters within lung adenocarcinoma samples. Gene expression levels are represented by a color gradient, with red indicating higher expression. **(B)** Volcano plot illustrating the differentially expressed genes between clusters, with statistically significant genes highlighted in green (downregulated) and red (upregulated), based on adjusted p-values and log fold changes. **(C)** Series of heatmaps representing consensus matrix scores for different cluster numbers, supporting the selection of the optimal number of clusters based on stability and consistency of the patterns shown. **(D)** The consensus cumulative distribution function (CDF) plot for various cluster numbers, assisting in determining the most stable cluster solution. **(E)** Delta area plot depicting the relative change in area under the CDF curve for different numbers of clusters (k), facilitating the identification of the cluster number with the highest stability. **(F)** Kaplan-Meier survival analysis comparing the survival probability between patients in different clusters, with the p-value indicating significant differences in survival outcomes.

**Supplementary Figure 4: Gene Expression Profiling and Functional Enrichment Analysis in Lung Adenocarcinoma.**

**(A)** Heatmap with hierarchical clustering based on gene expression data from lung adenocarcinoma samples, classified into distinct clusters. The color scale represents gene expression intensity, with red indicating high expression and blue indicating low expression. Clusters are color-coded in the column dendrogram. **(B)** Bar graph of the top enriched terms in the biological process category from the Gene Ontology (GO) analysis, showing the count of genes associated with terms related to chromosome segregation and mitotic nuclear division, among others. The color gradient represents the p-value, indicating the significance of the enrichment. **(C)** Bar graph of the top enriched pathways from the Kyoto Encyclopedia of Genes and Genomes (KEGG) analysis, with pathways such as cell cycle, p53 signaling, and DNA replication highlighted. The color intensity denotes the p-value, reflecting the significance of the pathway enrichment. KEGG pathway information was used for analysis.

**Supplementary Figure 5: Integrated analysis of differentiation states and survival correlations in cancer cohorts from TCGA and GEO databases. (A)**: Lasso coefficient profiles for genes across different levels of regularization indicated by log(lambda), illustrating the selection of features in the model. **(A)**: Visualization of the optimal lambda selection in the lasso model, with the lowest cross-validated error marked by the dashed vertical line. **(C, D)** : Heatmaps of expression data for the top differentially expressed genes from the TCGA experimental and validation cohorts, respectively, with expression levels color-coded from blue (low) to red (high). **(E)**: Heatmap of the top differentially expressed genes from the external GEO validation cohort, with the same color-coding as C and D. **(F, G)**: Kaplan-Meier curves for the TCGA cohorts, depicting survival probabilities for patients classified into high-risk (red) and low-risk (blue) groups based on their gene expression profiles. **(H, K)**: Kaplan-Meier survival plots for the GEO cohort, using the same risk score stratification as applied to the TCGA data. **(I, J):** Scatter plots indicating the distribution of risk scores in the TCGA experimental and validation cohorts, with patient survival status denoted by color: red for deceased and blue for alive. **(K):** Kaplan-Meier survival plot for the TCGA experimental cohort, illustrating the survival probability over time for patients stratified by risk score. **(L, M):** Kaplan-Meier survival analysis for the TCGA validation cohort, showing the survival probabilities for high-risk and low-risk groups based on gene expression profiles, with p-values indicating the statistical significance of the difference in survival**. (N):** Kaplan-Meier survival plot for the external GEO validation cohort, demonstrating the predictive power of the risk score on patient survival, with the survival curves for high-risk and low-risk groups and associated p-value.
